# Characterization of Selected Microalgae Species as Potential Sources of Nutrients and Antioxidants

**DOI:** 10.3390/foods13132160

**Published:** 2024-07-08

**Authors:** Natália Čmiková, Przemysław Łukasz Kowalczewski, Dominik Kmiecik, Aneta Tomczak, Agnieszka Drożdżyńska, Mariusz Ślachciński, Jakub Królak, Miroslava Kačániová

**Affiliations:** 1 Institute of Horticulture, Faculty of Horticulture and Landscape Engineering, Slovak University of Agriculture, Tr. A. Hlinku 2, 94976 Nitra, Slovakia; n.cmikova@gmail.com; 2 Department of Food Technology of Plant Origin, Poznań University of Life Sciences, 31 Wojska Polskiego St., 60-624 Poznań, Poland; dominik.kmiecik@up.poznan.pl; 3 Department of Biochemistry and Food Analysis, Poznań University of Life Sciences, 48 Mazowiecka St., 60-623 Poznań, Poland; aneta.tomczak@up.poznan.pl; 4 Department of Biotechnology and Food Microbiology, Poznań University of Life Sciences, 60-627 Poznań, Poland; agnieszka.drozdzynska@up.poznan.pl; 5 Institute of Chemistry and Technical Electrochemistry, Poznan University of Technology, 4 Berdychowo St., 60-965 Poznań, Poland; mariusz.slachcinski@put.poznan.pl; 6 Students’ Scientific Club of Food Technologists, Poznań University of Life Sciences, 31 Wojska Polskiego St., 60-624 Poznań, Poland; jakubkrolak1@wp.pl; 7 School of Medical and Health Sciences, University of Economics and Human Sciences in Warsaw, Okopowa 59, 01-043 Warszawa, Poland

**Keywords:** microalgae, protein content, amino acids, fatty acids, minerals, polyphenols, vitamin B

## Abstract

Microalgae are exceptional organisms from a nutritional perspective, boasting an array of bioactive compounds that have long justified their incorporation into human diets. In this study, we explored the potential of five microalgae species: *Nannochloropsis* sp., *Tetraselmis chuii*, *Chaetoceros muelleri*, *Thalassiosira weissflogii*, and *Tisochrysis lutea*. We conducted comprehensive analyses of their nutritional profiles, encompassing protein content, individual amino acid composition, mineral and trace element levels, fatty acid profiles (including saturated fatty acids (SFAs), monounsaturated fatty acids (MUFAs), and polyunsaturated fatty acids (PUFAs)), polyphenol compositions, and vitamin B content. The antioxidant activity of the ethanolic extracts was evaluated using two methods: ABTS and DPPH radical scavenging assay. The total protein content of the microalgae ranged from 34.09 ± 0.39% to 42.45 ± 0.18%, with the highest concentration observed in *T. weissflogii*. Essential amino acids such as histidine, threonine, lysine, valine, isoleucine, leucine, phenylalanine, and methionine were present in concentrations ranging from 0.53 ± 0.02 to 12.55 ± 2.21 g/16 g N. Glutamic acid emerged as the most abundant amino acid, with concentrations ranging from 6.73 ± 0.82 to 12.55 ± 2.21 g/16 g N. Among the microalgae species, *T. chuii* exhibited the highest concentrations of calcium (Ca) and manganese (Mn), while *C. muelleri* showed prominence in magnesium (Mg), sodium (Na), and iron (Fe). *T. weissflogii* stood out for its potassium (K) content, and *T. lutea* contained notable amounts of copper (Cu), zinc (Zn), and lead (Pb). Regarding fatty acid profiles, *Nannochloropsis* sp. and *T. chuii* were predominantly composed of SFA, while *C. muelleri* and *T. weissflogii* were rich in MUFA. PUFAs dominated the fatty acid profile of *T. lutea*, which also exhibited the most diverse range of polyphenolic substances. We also analyzed the B vitamin content, with *T. lutea* displaying the highest concentrations of niacin (B_3_) and riboflavin (B_2_). Antioxidant activity was confirmed for all microalgae tested using DPPH and ABTS radical IC_50_ (mg/mL) converted to Trolox equivalent (TEAC). These findings underscore the substantial potential of the examined microalgae species as sources of biologically valuable substances characterized by rapid growth and relatively undemanding cultivation conditions.

## 1. Introduction

One of the great challenges of the 21st century is the need to sustainably feed an ever-expanding world population, all within the constraints of increasingly limited natural resources [1]. Today, approximately one in nine people in the world is undernourished, with protein-energy malnutrition being critical [2].

The global demand for macroalgae (seaweed and kelp) and microalgae (unicellular algae) as food sources is growing. Algae consumption is expanding beyond traditional aspects of nutrition and health due to their functional benefits [3]. The abundance of bioactive compounds found in microalgae has long driven their utilization in human nutrition [4].

Unlike traditional crops, microalgae bring unique advantages because they thrive without the need for arable land, in seawater, and use residual nutrients. Their remarkable areal productivity and rich composition of oils, proteins, and carbohydrates further highlight their potential to address a variety of needs. Through the use of biorefinery techniques, the harvested microalgae biomass can be carefully fractionated, offering a versatile range of food and non-food products [5,6].

Microalgae, with a history spanning centuries, have entered the realm of large-scale commercial production in recent decades. These versatile organisms can thrive in diverse environments, from open-culture systems like lakes to meticulously controlled close-culture systems. Demonstrating superior productivity compared to traditional crops, microalgae can flourish in climatic conditions and regions unsuitable for conventional crops, including arid deserts and coastal areas [7].

Seaweed proteins find applications in a variety of industries, including food products, animal feed and aquaculture, nutritional supplements, pharmaceuticals, and cosmetics. However, the exploration and utilization of microalgae in human food is still in the early stages, suggesting a promising but developing area within this field [8,9,10,11].

Beyond human consumption, microalgae play a crucial role in feeding various animals, spanning from household pets like cats and dogs to aquarium and ornamental fish, birds, horses, poultry, cows, and breeding bulls. Furthermore, a spectrum of microalgae, including *Tetraselmis*, *Isochrysis*, *Pavlova*, *Phaeodactylum*, *Chaetococeros*, *Nannochloropsis*, *Skeletonema*, and *Thalassiosira*, finds application as feeds in aquaculture [12,13]. In addition to their importance in aquaculture, microalgae have gained attention as prime candidates for “nutraceuticals” or “functional foods” due to their remarkable ability to synthesize valuable components such as carotenoids, long-chain fatty acids, essential and non-essential amino acids, enzymes, vitamins, and minerals. Matos et al. [14] identified algae as having significant potential for human nutrition, contributing to the growing recognition of microalgae as key resources in health and well-being.

The substantial protein content found in numerous microalgal species, exemplified by figures such as 55–70% for *S. platensis* and 42–55% for *C. vulgaris* (values per dry matter), stands as a pivotal factor driving the recognition of these organisms as a viable source of food [13]. The amino acid profile of almost all algae matches the requirements set by the Food and Agriculture Organization (FAO). Minor deficiencies are observed for sulfur-containing amino acids, namely methionine and cysteine, a characteristic shared with many plant proteins [15,16]. In the study by Santiago-Díaz et al. [17], the proportions of amino acids in the dry biomass of selected algae were analyzed. The authors reported a high content of essential amino acids, with values reaching 54.1%, 61.2%, and 72.6% in *Chloromonas* cf. *reticulata*, *Chloroidium saccharophilum,* and *Pseudopediastrum boryanum*, respectively. Among all the microalgal samples, glutamic acid emerged as the most abundant free amino acid. In *C. saccharophilum* and *P. boryanum*, proline and lysine followed as the next most abundant, while in *C. reticulata*, it was methionine and then lysine.

The mineral content of seaweed is typically higher compared to terrestrial plants and animal products. Ranging from 8 to 40%, it includes essential minerals and trace elements important for human nutrition. Consequently, edible seaweeds are proving to be important sources of minerals, particularly as some of the trace elements essential for nutrition are either absent or present in minimal amounts in terrestrial vegetables [18,19,20,21,22].

Lipids emerge as the compounds most commonly extracted from microalgae, demonstrating significant potential for commercialization. The diverse composition of microalgal lipids, encompassing the unique lipid and fatty acid (FA) profiles attributed to the species diversity of microalgae, represents a distinctive and promising natural resource. Microalgae exhibiting substantial lipid synthesis are particularly regarded as a valuable and prospective raw material [23,24].

According to some studies, microalgae may exhibit phenolic levels equal to or lower than the minimum reported levels in terrestrial plants. The algal content of phenolics consists primarily of phenolic acids. However, recent investigations have revealed a more diverse range of phenolic compounds in microalgae, including different classes of flavonoids, such as isoflavones, flavanones, flavonols, and dihydrochalcones. This suggests the ability of microalgae to produce complex phenolic compounds, highlighting the need for comprehensive characterization and identification of these chemicals, especially given the potential existence of novel phenolics [25,26,27,28].

Vitamins, essential for the maintenance of life, are either not synthesized or synthesized in limited amounts in animals and humans. Their continuous intake from dietary sources, such as plants, fruits, or seeds, is required. Nevertheless, not all plants contain all types of vitamins. Some vitamins, such as D, K, or specific B vitamins, are found in insufficient amounts. Microalgae serve as a valuable source of essential vitamins for humans and surpass terrestrial plants in their ability to provide certain vitamins. In particular, microalgae offer access to vitamins D and K, which are often scarce in many terrestrial plants or fruits. Similarly, microalgae are a rich source of several B vitamins, including B_12_, B_9_, and B_6_, while also providing other key vitamins such as A, C, and E. The diverse range of vitamins found in the cells of microalgae makes these organisms promising natural vitamin producers for human consumption [29,30,31].

Foods of plant origin contain almost all the essential mineral and organic nutrients needed for human nutrition, together with a number of characteristic organic phytochemicals that contribute to overall health [32]. Protein plays several important roles in the human diet, in addition to the primary function of supplying amino acids necessary for human nutrition [33]. Fatty acids are necessary to maintain the composition, integrity, and functionality of membranes. This highlights the importance of providing an appropriate balance to cells and tissues to achieve optimal performance [34]. Minerals play diverse roles and offer potential benefits to the metabolism and balance of the body, including fortifying bones, synthesizing hormones, transmitting nerve signals, and serving as essential components of numerous enzymes [35]. Physiologically, phenolic compounds play a key role in defense mechanisms, exhibiting their anti-aging, anti-inflammatory, antioxidant, and anti-proliferative activities. These compounds help to reduce the risk of certain chronic diseases, such as diabetes, cancer, and cardiovascular disease [36]. B vitamins are obtained primarily from daily food intake. These nutrients act as cofactors and facilitate various metabolic processes in the human body [37].

In addition, algae can biosynthesize a number of bioactive substances, including polyphenols, triterpenoids, polyunsaturated fatty acids, and polysaccharides. Due to the presence of this bioactive diversity, microalgae have a wide range of biological capabilities, including antioxidant activities [38].

In this research, the potential of microalgae as an innovative ingredient that offers a range of health benefits to humans was investigated. Despite their promise, microalgae and their nutritional potential have not been sufficiently studied so far. To address this gap, we analyzed five commercially available microalgal powders obtained from *Nannochloropsis* sp., *Tetraselmis chuii*, *Chaetoceros muelleri*, *Thalassiosira weissflogii*, and *Tisochrysis lutea*. These algae were grown under optimized conditions to ensure their consistently high quality. The algae and their extracts were tested for total protein content, amino acid profiles, mineral composition, fatty acids, and vitamin B content. This was conducted in order to contribute further information and enhance the understanding of the composition and health benefits of microalgae.

## 2. Materials and Methods

### 2.1. Samples

Powdered *Nannochloropsis* sp., *Tetraselmis chuii*, *Chaetoceros muelleri*, *Thalassiosira weissflogii*, and *Tisochrysis lutea* were purchased from the company Proviron (Hemiksem, Belgium). The company declares that the microalgae are produced under the most strictly controlled conditions and continuously checked for the presence of pathogens (Hazard Analysis and Critical Control Points and Food Contact Materials certified by Société Générale de Surveillance (FCA certificate BE 01/1522.GF)). All samples were purchased in 2022 and stored hermetically sealed in a dark and dry environment at room temperature (~20 °C).

### 2.2. Protein Content

The protein content was determined using the Kjeldahl method according to ISO 20483:2013 [39]. The samples were subjected to digestion with sulfuric acid in the presence of a catalyst. The resulting reaction products were made alkaline and then distilled. The ammonia released was collected in a boric acid solution, and the solution was diluted with a sulfuric acid solution. The aim of this procedure is to determine the nitrogen content, which facilitates the calculation of crude protein content.

### 2.3. Amino Acid Composition

Protein hydrolysis was performed using two independent methodologies: acidic (110 °C, 23 h) and oxidative (4 °C, 16 h and 100 °C, 2 h)—official AOAC method 994.12 [40]. The use of acidic hydrolysis enables the determination of most protein amino acids: L-alanine (Ala), L-arginine (Arg), L-aspartic acid (Asp) + L-asparagine (Asn), L-glutamic acid (Glu) + L- Glutamine (Gln), L-leucine (Leu), L-lysine (Lys), L-serine (Ser), L-threonine (Thr), L-tyrosine (Tyr), L-valine (Val), L-histidine (His), L-Isoleucine (Ile), L-Phenylalanine (Phe), L-Proline (Pro), and Glycine (Gly). Oxidative hydrolysis allows the determination of sulfur-containing amino acids: L-methionine (Met) and L-Cystine (Cys). The amino acid contents were subsequently determined employing the methodology described by Tomczak et al. [41]. The amino acid dilution and derivatization procedure were conducted using AccQ•Tag reagents (No. 186003836, Waters Corporation, Milford, MA, USA) following the manufacturer’s instructions subsequent to sample evaporation. Ultra-efficient liquid chromatography (UPLC) analysis was carried out using a Shimadzu Nexera 2.0 system equipped with a binary solvent manager, autosampler, column heater, and PDA detector (Kyoto, Japan). Separation was achieved using an AccQ-Tag Ultra C18 1.7 μm column (2.1 mm i.d. × 100 mm, 1.7 μm particles, Waters) with a mobile phase flow rate of 0.6 mL/min and a column temperature of 55 °C. A non-linear gradient mixing 5% and 100% AccQ•Tag Ultra solvent was used. Detection was performed at 260 nm with a PDA detector set at a sampling rate of 20 points/sec. Quantification of amino acids utilized standards containing 2.5 μmol/mL for each amino acid in 0.1 mol/L HCl (088122, Waters), which were diluted 25 times with ultrapure water. The diluted standard (10, 20, or 60 μL) was mixed with 70 μL of borate buffer and 20 μL of AccQ•Tag reagents for standard amino acid derivatization. UPLC analysis of the sample was conducted using 1 μL injection volume, repeated 5 times to establish a calibration curve with the LabSolution program (Shimadzu Corp., Kyoto, Japan). Amino acid content is expressed as g/16 g N (equivalent to g/100 g protein).

### 2.4. Determination of Mineral Profile

An innovative high-pressure/high-temperature system utilizing concentrated microwave energy was employed to mineralize the algae powders. The samples were introduced into sealed vessels of 30 mL capacity, which were crafted from chemically modified Teflon (Hostaflon TFM, Hoechst AG, Frankfurt/Main, Germany). Subsequently, 3 mL of 60% nitric acid and 1 mL of 30% hydrogen peroxide were introduced into these vessels. The assembly was encased in a steel jacket, wherein microwave energy, supplied by an antenna with a power of 200 W, facilitated a 10 min-long mineralization process. Following the mineralization, the samples underwent dilution to a final volume of 25 mL.

To analyze the elemental content utilizing the ICP OES technique, an emission spectrometer with an excitation source of inductively coupled plasma (IRIS HR, Thermo Jarell Ash, Waltham, MA, USA) was employed. Quantitation was conducted utilizing the calibration curve technique. The contents of calcium (Ca), magnesium (Mg), potassium (K), sodium (Na), copper (Cu), iron (Fe), manganese (Mn), zinc (Zn), and lead (Pb) were determined. The results, expressed in milligrams per gram of dry matter (mg/g dm), were obtained on the basis of six independent readings—three biological and two technical replicates.

### 2.5. Fatty Acids Composition

Fatty acid extraction was performed according to an established protocol described in detail by Folch et al. [42]. Fatty acid composition was determined according to the official AOCS Ce 1 h-05 method [43] using parameters detailed in previous literature [44]. An Agilent 7820A gas chromatograph (Agilent Technologies, Santa Clara, CA, USA) equipped with a flame ionization detector (FID) and an SLB-IL111 (100 m, 0.25 mm, and 0.20 μm) capillary column (Supelco, Bellefonte, PA, USA) was used. The results were expressed as percentages of the total fatty acid content.

### 2.6. Methanolic Extraction

Methanolic extracts were prepared from the algae powders. The extraction process involved suspending the sample in an 80% methanol solution in water (*v*/*v*). A ratio of algae to solvent of 1:10 was used. The mixture was vigorously shaken using a laboratory shaker S50 (CAT Germany GmbH, Zülpich, Germany) for 60 min. Following this, the samples underwent centrifugation at 12,000× *g* for 15 min at 4 °C. The resulting supernatants were carefully decanted, filtered through a 0.45 µm PTFE syringe filter, and then stored in a glass flask at −20 °C prior to the analyses.

### 2.7. Polyphenols Profile Composition

The determination of polyphenolic compounds through high-performance liquid chromatography (HPLC) was conducted using an Agilent 1260 Infinity II liquid chromatograph (Agilent Technologies, Inc., Santa Clara, CA, USA) according to the method described by Drożdżyńska et al. [45]. The instrumentation included an autosampler (G7129A), a pump (G7111A), and a diode detector (G7115A) with a spectrum overview spanning from 190 to 400 nm. The separation of phenolic compounds was accomplished using an SB-C18 column (50 mm × 4.6 mm with 1.8 µm particle diameter, Agilent) maintained at a temperature of 25 °C.

Elution was achieved using solvents A (water and acetic acid, 98:2 by volume) and B (methanol and acetic acid, 98:2 by volume). The elution utilized the following gradient profile: 0 min at 2% B, 22 min at 40% B, 26 min maintained at 40% B, 28 min at 100% B, and finally, at 36 min returning to 2% B. The flow rate was 0.75 mL/min. A sample volume of 5 µL was injected into the column. Quantitative calculations were performed on the basis of peak areas using OpenLab CDS (Agilent Technologies, Inc., Santa Clara, CA, USA). The results were expressed as micrograms per gram of dry matter (µg/g dm).

### 2.8. Analysis of B Vitamins

Vitamins of the B class were analyzed according to the method proposed by Li and Chen [46] using an Agilent 1260 Infinity II liquid chromatograph equipped with a G7129A automatic sample feeder, G7111A pump, and G7115A DAD WR detector. The spectral range for analysis spanned from 210 to 400 nm. Extracts obtained according to the method described in Section 2.6 were subjected to analyses performed using a Lichrospher^®^RP-18e column (5 μm, 250 × 4 mm, Merck, Darmstadt, Germany).

Elution was performed at a flow rate of 1 mL/min, utilizing a mobile phase composed of 0.1 M KH_2_PO_4_ at pH 7 (phase A) and acetonitrile (phase B). The elution followed a gradient starting at 0 min with 3% B, progressing to 10% B at 15 min, reaching 30% B at 40 min, maintaining 30% B until 45 min, reverting to 3% B at 46 min, and stabilizing at 3% B until 56 min. The chromatographic analysis was conducted at a temperature of 30 °C, and the samples were introduced into the column in a volume of 10 µL.

### 2.9. Antioxidant Activity

The antioxidant activity of ethanolic algae extracts was measured using the radical scavenging assays 2,2-diphenyl-1-picrylhydrazyl (DPPH) and 2,2′-azino-bis(3-ethylbenzothiazoline-6-sulfonic acid) (ABTS), both obtained from Sigma-Aldrich, Taufkirchen, Germany. The algae extracts were dissolved in 100% DMSO to a concentration of 50 mg/mL. DPPH was prepared in methanol at a concentration of 0.025 g/L, with its absorbance adjusted to 0.8 at 515 nm, using a Glomax spectrophotometer from Promega Inc., Madison, WI, USA. The ABTS radical cation was generated following a previously described method and diluted to an absorbance of 0.7 at 744 nm before analysis [47]. For the assays, 190 μL of either the DPPH or ABTS solution was mixed with 10 μL of the algae extracts in a 96-well microtiter plate and incubated for 30 min with continuous shaking at 1000 rpm at room temperature in the dark. Absorbance decreases at 744 nm and 515 nm were recorded for the ABTS and DPPH assays, respectively. The percentage inhibition of DPPH or ABTS was calculated using the formula (A_0_ − A_A_)/A_0_ × 100, where A_0_ was the absorbance of DPPH or ABTS with methanol, and A_A_ was the absorbance of the sample. Trolox, dissolved in methanol (Uvasol^®^ for spectroscopy, Merck, Darmstadt, Germany) to a concentration range of 0–100 µg/mL, was used as a standard reference to calculate the total antioxidant capacity, expressed according to the Trolox calibration curve (TEAC).

### 2.10. Statistical Analysis

Unless stated otherwise, all analyses were run in triplicate. Mean values are reported with the corresponding standard deviation values (SD). One-way ANOVA Tukey’s test at a significance level of *p* ≤ 0.05 was performed in Statistica v13.3 (TIBCO Software Inc., Palo Alto, CA, USA).

## 3. Results and Discussion

### 3.1. Protein and Amino Acid Content

The total protein content present in the microalgae ranged from 34.09 ± 0.39% to 42.45 ± 0.18% (Table 1). The lowest percentage of protein was found in *C. muelleri* (34.09 ± 0.39%) and the highest in *T. weissflogii* (42.45 ± 0.18%). The other microalgae showed protein percentages ranging from 39.48 ± 1.80 to 41.68 ± 0.58%.

Microalgae are widely recognized as abundant sources of protein [48]. Their protein content is influenced by various factors. In a study conducted by Hulatt et al. [49], the growth of *Nannochloropsis* sp. in flat photobioreactors was optimized for both fatty acid and protein production. The experiment involved eighteen cultivations at two different nutrient concentrations, with analyses of fatty acids, protein content, and caloric values conducted at intervals of 8, 12, and 16 days. The highest protein content was observed during nutrient-rich growth, with proteins constituting 54.9 ± 1.70% of the total dry matter. The lowest protein content recorded in the study was only 24.6 ± 1.50%. This underscores the significant influence of environmental conditions during the growth and harvesting phases on the protein content of algae. This statement is further supported by the results of Rebolloso-Fuentes et al. [50], who tested the composition of *Nannochloropsis* sp. cultured in an indoor chemostat under continuous illumination. On average, the biomass contained 28.80% crude protein. The nutrient composition of the biomass was significantly affected by the residence time in the photobioreactor. Biomass harvested when the residence time was short was richer in protein and eicosapentaenoic acid than the one collected at a long residence time. In a study by Khatoon et al. [51], the composition of microalgae cultivated in wastewater was studied. An increased protein content in *T. chuii* (56.40% of dry weight) was observed in these conditions. This is yet another indication suggesting that the protein content of microalgae can vary considerably with growth conditions. Brown [52] tested the composition of sixteen species of microalgae. All cultures were grown under standard conditions and harvested at a defined growth stage. The protein content of *T. chuii* was determined to be 31%. Contrastingly, algae of the same species purchased for our study were grown under optimized conditions, and the protein content was greater by up to 10%. Xin-Wei Wang [53] cultivated *C. muelleri* under conditions of increasing CO_2_ content: air (0.03% CO_2_), 10%, 20%, and 30%. The reported protein content ranged from 19.58% to 37.48%. The results of our study (34.09 ± 0.39%) fit within this range. All of the algae tested in our study were purchased from a company that declares that the microalgae are grown using fully controlled methods optimized for the best nutritional properties, which include protein content. In general, it can be concluded that our results for total protein content were similar or higher than the ones reported in the referenced studies. 

Microalgae can also be compared to other protein-rich food ingredients. The protein content determined for the powders investigated in this study (34.09 ± 0.39 to 42.45 ± 0.18%) was significantly higher than the one reported by Iqbal et al. [54], where lentils proved to be a legume with the highest protein content of 26.1%, and were closely followed by green peas (24.9%). Nuts are also high in protein, and Cho [55] found that the protein content of different nuts ranged from 22.54% to 25.42%, which is lower than that of microalgae as well.

**Figure 1:**
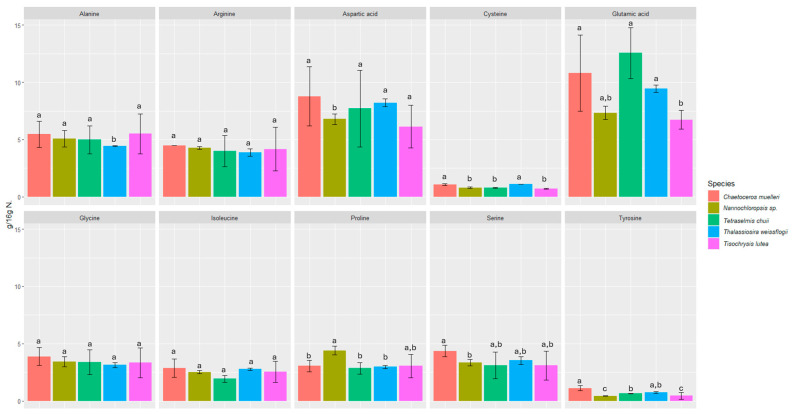
Content of non-essential amino acids in microalgae expressed in (g/16 g N). Values marked with the same letter for amino acids are not significantly different, *p* > 0.05.

**Figure 2:**
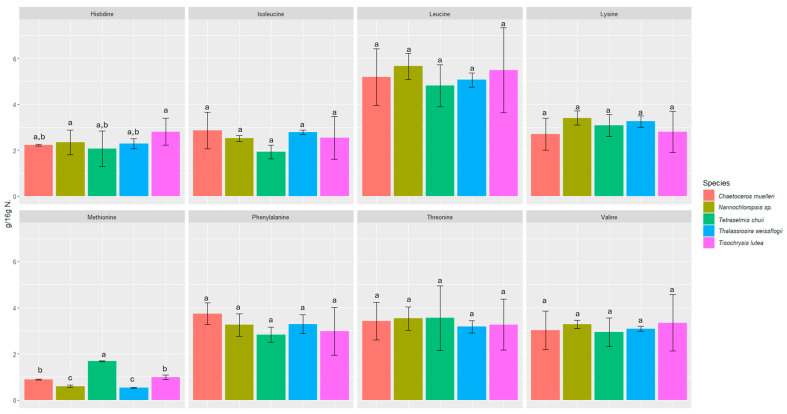
Content of essential amino acids in microalgae expressed in (g/16 g N). Values marked with the same letter for amino acids are not significantly different, *p* > 0.05.

We investigated the content of seventeen amino acids in selected microalgae, including eight essential amino acids (Figure 1 and Figure 2).

All the microalgae contained essential amino acids such as histidine, threonine, lysine, valine, isoleucine, leucine, phenylalanine, and methionine in amounts ranging from 0.53 ± 0.02 to 12.55 ± 2.21 g/16 g N. The amounts of each amino acid were fairly consistent among all the microalgae samples studied, and in all of them, glutamic acid was the most abundant (6.73 ± 0.82 to 12.55 ± 2.21 g/16 g N).

The biochemical profiles of 16 species of microalgae commonly used in mariculture were analyzed in detail by Brown [52]. Considerable variability in protein content was observed between these species. The amino acid composition was relatively similar, but *T. chui* and *T. suecica* were richer in arginine, while *N. atomus* showed increased proline content. The levels of essential amino acids in microalgae were either comparable to or exceeded those in oyster larvae, which reflects the high quality of protein in all the tested microalgal species. Our study also confirmed the presence of proline (2.85 ± 0.51–4.41 ± 0.39 g/16 g N) and arginine (3.88 ± 0.31–4.47 ± 0.01 g/16 g N). In a study by Brown and Jeffrey [56], minimal differences were observed in amino acid profiles between species, with the exception of tryptophan and arginine, for which more pronounced differences were noticed. In our study, the differences between species were also minimal in this respect. However, the highest statistically significant difference was found for tyrosine and methionine. The most abundant amino acids were glutamic acid (6.73 ± 0.82 to 12.55 ± 2.21 g/16 g N), aspartic acid (6.14 ± 1.86 to 8.78 ± 2.59 g/16 g N), leucine (4.81 ± 0.91 to 5.66 ± 0.57 g/16 g N), and alanine (4.43 ± 0.05 to 5.51 ± 1.75 g/16 g N). L-alanine and glutamic acid represented the dominant components of free amino acid content in most of the species analyzed in a study by Araya et al. [57]. Among the species studied, *Haematococcus pluvialis* showed the lowest concentration of free amino acids, reaching 38.8 ± 0.15 mg AA/100 g dry weight (DW). The primary amino acids identified in the study by Tibbetts et al. [58] were aspartic acid and glutamic acid (as in our study), which accounted for a predominant proportion in the range of 20–30% protein (8–12% of DW). These findings reinforce the consensus that the amino acid composition of microalgal biomass is not only comparable to but often exceeds that of traditional plant protein sources.

### 3.2. Mineral and Trace Element Content

The tested group of microalgae was heterogeneous in terms of mineral and trace element composition (Table 2). *T. chui* was the most abundant of all in calcium (54,900.00 ± 4390.00 µg/g) and manganese (139.00 ± 11.00 µg/g); *C. muelleri* contained the highest amounts of magnesium, sodium, and iron (7560.00 ± 600.00; 73,000.00 ± 5800.00; 969.00 ± 78.00 µg/g); *T. weissflogii* contained the highest amount of potassium (34,700.00 ± 2800.00 µg/g); *T. lutea* contained the richest in three trace elements tested—copper (78.50 ± 6.30 µg/g), zinc (181.0 ± 14.00 µg/g), and lead (108.0 ± 9.00 µg/g). In the case of no mineral or trace element, *Nannochloropsis* sp. showed the highest concentration.

Sparse information exists regarding the elemental composition of microalgal biomass. In contrast, numerous species of macroalgae (seaweed) have undergone comprehensive characterization. This discrepancy could have been somewhat anticipated. While microalgae may contain certain elements of nutritional significance, their inorganic elemental composition (ash) is typically considerably lower than that of macroalgae. The ash content in microalgae (excluding diatoms) ranges from 4% to 20%, whereas in macroalgae, it can reach 22% to 64% [56,57,58]. It is known, however, that both macroalgae and microalgae are important sources of minerals. The results of our study confirm it, as does the report by Tokuşoglu et al. [59]. In the referenced research, it was found that spirulina contains a significant amount of K, chlorella is rich in P, and *Isochrisis* is an important source of Ca and Mg. In addition, the Se content of *T. lutea* was found to exceed that of other microalgae. The nutritional composition of *Nannochloropsis* sp. was analyzed in a study by Rebolloso-Fuentes et al. [50]. The concentrations of minerals in 100 g of dry biomass were as follows: Ca (972 mg), K (533 mg), Na (659 mg), Mg (316 mg), Zn (103 mg), Fe (136 mg), Mn (3.4). mg), Cu (35.0 mg), Ni (0.22 mg), and Co (<0.1 mg). The contents of toxic heavy metals (Cd and Pb) were negligible, as the microalga was cultured in an indoor chemostat under continuous illumination. In our research, *Nannochloropsis* sp. was found to contain approximately twice as much sodium and potassium. Specifically, sodium levels were 13,900.00 ± 1100.00 µg/g, translating to 1390.00 ± 110.00 mg/100 g, and potassium levels were 10,100.00 ± 800.00 µg/g, translating to 1010.00 ± 80.00 mg/100 g. 

### 3.3. Fatty Acid Composition

**Figure 3:**
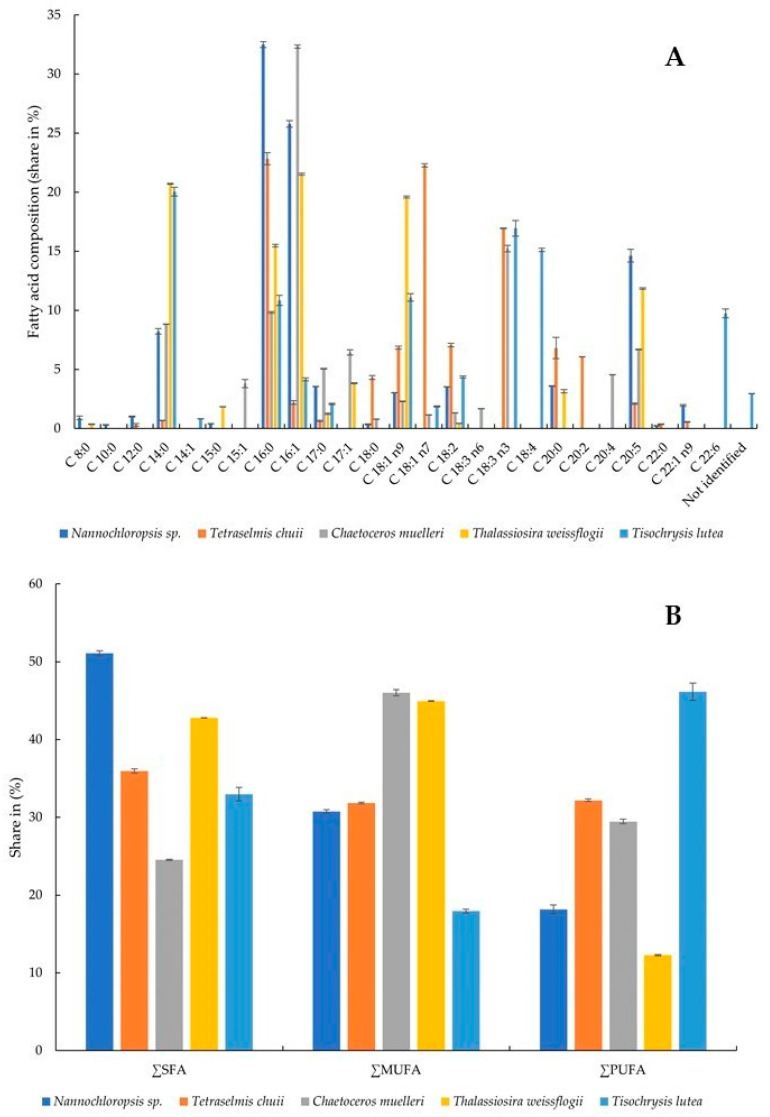
Fatty acid composition (share in %) (**A**) and share of SFA, MUFA, and PUFA (**B**): SFA, saturated fatty acid; MUFA, monounsaturated fatty acid; PUFA, polyunsaturated fatty acid.

The presence and relative amounts of selected fatty acids were determined in the microalgae. All of the tested fatty acids were present in the samples, although not all of them simultaneously (Figure 3A,B). The highest share of C 8:0 (caprylic acid) was recorded in *Nannochloropsis* sp. algae (0.90 ± 0.15%) and *T. weissflogii* (0.36 ± 0.02%). This acid was not detected in the other samples. C 10:0 (capric acid) was not detected in any algae except *Nannochloropsis* sp. (0.31 ± 0.03%). C 12:0 (lauric acid) was present in two samples, although at significantly different levels: *Nannochloropsis* sp. (1.01 ± 0.05%) and *T. chuii* (0.31 ± 0.10%). C 14:0 (myristic acid) was present in all algae, and its percentages ranged from 0.69 ± 0.01 to 20.71 ± 0.05% in *T. chuii and T. weissflogii*, respectively. C 14:1 (myristoleic acid) was detected only in *T. lutea* at a level of 0.82 ± 0.02%. C 15:0 (pentadecanoic acid) was present in two algae—*Nannochloropsis* sp. (0.42 ± 0.01%) and *T. weissflogii* (1.85 ± 0.01). C 15:0 was found in *T. weissflogii* at a level of 1.85 ± 0.01%. C 16:0 (palmitic acid) was present in all algae at levels ranging from 9.83 ± 0.07% (*C. muelleri*) to 32.50 ± 0.25% (*Nannochloropsis* sp.). So was C 16:1 (palmitoleic acid), in which case the highest amount was found in *C. muelleri* (32.33 ± 0.14). The alga of this species also contained the highest levels of C 17:0 (margaric acid) and C 17:1 (heptadecenoic acid) (5.06 ± 0.03% and 6.44 ± 0.22%, respectively). C 18:0 (stearic acid), C 18:1 n7 (vaccenic acid, monounsaturated omega-7), C 18:3 n3 (α-linolenic acid, polyunsaturated omega-3), C 20:0 (arachidic acid), and C 20:2 (eicosadienoic acid) were present in the highest share in the alga *T. chuii*. Powdered *C. muelleri* contained the highest amount of C 18:3 n6 (γ-linolenic acid, polyunsaturated omega-6) and C 20:4 (arachidonic acid, polyunsaturated omega-6). C 18:1 n9 was present in all algae, with the largest content determined in *T. weissflogii*. *T. lutea* was the richest of all in C 18:2 (linoleic acid, polyunsaturated omega-6), C 18:3 n3 (α-linolenic acid, polyunsaturated omega-3), and C 22:6 (docosahexaenoic acid, polyunsaturated omega-3). C 20:5 (eicosapentaenoic acid, polyunsaturated omega-3) and C 22:1 n9 (erucic acid, monounsaturated omega-9) were present in the highest amounts in the algae *Nannochloropsis* sp. In general, *Nannochloropsis* sp. had the highest content of saturated fatty acids (SFAs) at 51.075 ± 0.349%, while *C. muelleri* had the lowest content (24.524 ± 0.092%). Monounsaturated fatty acids (MUFAs) were quantified in the highest amount in the microalga *C. muelleri* (46.022 ± 0.389%) and the lowest in *T. lutea* (17.935 ± 0.242%). At the same time, *T. lutea* was found to contain 46.145 ± 1.094% polyunsaturated fatty acids (PUFAs), with 2.956 ± 0.014% fatty acids not identified. The lowest amount of PUFAs was found in the alga *T. weissflogii* (12.268 ± 0.077%).

The capacity of microalgae to accumulate lipids of differing composition has significant practical implications. Microalgae that can synthesize substantial quantities of lipids are regarded as a promising and valuable natural resource [60]. The profiles of fatty acids of six species of marine microalgae commonly used in aquaculture were studied by Servel et al. [61]. PUFAs constituted a significant part of total lipids in *Tetraselmis suecica*, *Porphyridium cruentum*, and *Isochrysis galbana*, which amounted to 20.9%, 17.1%, and 17%, respectively. Within *T. suecica*, arachidonic and linolenic acids were found to be the most abundant PUFAs. Meanwhile, *Skeletonema costatum*, *Chaetoceros calcitrans*, *P. cruentum*, and *Nannochloropsis* sp. showed high shares of eicosapentaenoic acid. *I. galbana* showed remarkable levels of linolenic, octadecatetetraenoic, and docosahexaenoic acids. Among the acids studied by us, the content of PUFAs ranged from 12.268 ± 0.077 in *T. weissflogii* to 46.145 ± 1.094% in *T. lutea*. Moreover, in the latter, the highest contents of linoleic acid (4.36 ± 0.08%), α-linolenic acid (16.94 ± 0.66%), and docosahexaenoic acid (9.74 ± 0.38%) among all the algae tested were found. In another study, the lipid content of 14 microalgal species was determined to range from 2.52 ± 0.03% to 14.05 ± 0.14% on a DW basis [62]. The lipids of microalgae consist predominantly of unsaturated fatty acids, the content of which ranges from 50% to 65%. Noteworthy, there is also a remarkably high abundance of palmitic acid (C16:0), which ranges from 17% to 40%. Linolenic (C18:3) and PUFA contents also deserve special attention [63]. In our study, *Nannochloropsis* sp. and *T. chuii* showed the highest content of SFAs, *C. muelleri* and *T. weissflogii*—the highest content of MUFAs, and only in *T. lutea* were PUFAs the most abundant.

### 3.4. Composition of Polyphenols

To further characterize the microalgae, the profiles of polyphenols were analyzed in order to further analyze their bioactive potential (Table 3). Of the eight substances tested, *Nannochloropsis* sp. contained three polyphenolic substances: kaempferol (1.47 ± 0.02 μg/g), vitexin (1.26 ± 0.03 μg/g), and *p*-hydroxybenzoic acid (2.94 ± 0.07 μg/g). *T. chuii* also contained three: kaempferol with 3.35 ± 0.05 μg/g, rutin (1.03 ± 0.013 μg/g), and *p*-hydroxybenzoic acid (1.61 ± 0.10 μg/g). *C. muelleri* did not contain any of the phenolic compounds tested. In *T. weissflogii*, kaempferol (0.99 ± 0.01 μg/g) and *p*-hydroxybenzoic acid (10.96 ± 0.11 μg/g) were present. *T. lutea* contained the highest number of substances under investigation, namely *p*-coumaric acid, catechin, chlorogenic acid, and *p*-hydroxybenzoic acid (1.07 ± 0.05; 0.36 ± 0.06; 0.79 ± 0.05; 7.28 ± 0.12 μg/g, respectively). Chlorogenic and gallic acids were not detected in any of the tested microalgae.

Microscopic algae are capable of producing polyphenolic compounds, although in limited quantities. The presence of numerous phenolic groups in molecules, such as flavonoids, allows efficient binding of heavy metal ions. This binding contributes to the accumulation of divalent metals in cells, while the extracellular forms participate in the chelation of heavy metals, ultimately reducing their toxicity. Phenolic compounds play a key role in the antioxidant protection of algae and contribute to the generation of an adaptive response to oxidative stress [64]. Phenolic acid was studied in *Spirulina* sp. and *Nannochloropsis* sp. in a study by Scaglioni et al. [65]. The authors analyzed their content in several fractions—soluble in methanol, soluble in ethanol, and bound. Gallic acid was present in both *Spirulina* sp. and *Nannochloropsis* sp. but only in the methanol extracts. The presence of coumaric acid was not confirmed in *Nannochloropsis* sp., similar to our results. Among the algae tested by us, the presence of coumaric was confirmed only in *T. lutea* (0.36 ± 0.06 µg/g). Chlorogenic acid was not found in any of the samples in our tests. In the case of the above-referenced study, this acid was only detected in the methanolic extract of *Spirulina* sp., where it was present in 585.20 µg/g. The presence of p-hydroxybenzoic acid was confirmed in all the microalgae tested by us (1.61 ± 0.10 µg/g–10.96 ± 0.11 µg/g), except *C. muelleri*. In the study by Scaglioni et al. [65], hydroxybenzoic was found in the methanol fraction (26.80 µg/g), the ethanol fraction (21.20 µg/g), but also in the bound (21.60 µg/g) form in *Nannochloropsis* sp. This acid was also found in methanolic extract (24.6 µg/g) and bound form (11.8 µg/g) in *Spirulina* sp. In a study by Haoujar et al. [66], the polyphenolic constituents were analyzed in the microalgae *Nannochloropsis* sp. Kaempferol was present in the algae at 12.10 ± 1.32 µg/g, whereas in our study, the quantified amount was multifold lower (1.47 ± 0.02 µg/g). In the same study, catechin content was determined at levels of up to 33.47 ± 3.14 µg/g, while it was not detected in our study. With the exception of *C. muelleri, p*-hydroxybenzoic acid (*p*-HBA) was detected in all the algae examined by us, with its concentrations ranging from 1.61 ± 0.10 to 10.96 ± 0.11 μg/g. This compound, recognized as a functional and regulatory substance, akin to phytohormones, draws particular attention. Intriguingly, *p*-HBA finds application as a bacteriostatic agent for food preservation, as it exhibits potent inhibitory effects on the growth of pathogenic bacteria, such as *E. coli* and *S. aureus*. The inclusion of trace amounts of *p*-HBA allows effective control of bacterial infection and mitigation of contamination risks [67,68].

### 3.5. Content of B Vitamins

The amounts of B vitamins, specifically niacin (B_3_), riboflavin (B_2_), and folic acid (B_9_), were also determined (Table 4). Niacin was found in all the tested samples; riboflavin was only found in four of them—it was not present in *T. weissflogii*. The highest contents of niacin and riboflavin were found in *T. lutea* (514.82 ± 2.95 and 20.44 ± 1.96 μg/g, respectively). Folic acid was detected only in *C. muelleri* (10.53 ± 0.92 μg/g). 

Vitamin deficiency in the human population is a global concern, necessitating effective solutions. Notably, among the diverse array of compounds present in microalgae, vitamins are of particular significance [29]. Microalgae show significant capacity to accumulate vitamins B_2_ and B_3_. In *Chlorella*, the highest measured concentrations of vitamin B_9_ were found to range from 3.10 to 34 μg/g DW. *Picochlorum* sp. showed a high vitamin B_9_ content of 64.70 μg/g DW, and in *Michrochloropsis*, a content of 43.60 μg/g DW was determined [29,69,70]. In a study by Edelmann et al. [69], the content of riboflavin was found to show minimal differences in different samples of *Spirulina* powder. On the contrary, significant variations were observed in riboflavin content between samples of powdered *Chlorella* (20.70 to 33.60 μg/g). In the only *N. gaditana* powder examined, a riboflavin content of 22.10 μg/g was determined. This was lower than the average values found in *Spirulina* (36.30 μg/g) and *Chlorella* (28.0 μg/g). These values are significantly higher than the ones determined for the samples of *Nannochloropsis* sp., *T. chuii*, *C. muelleri*, and *T. weissflogii* tested by in our study. Comparable values were only found in *T. lutea*, with a riboflavin content of 20.44 ± 1.96 μg/g. The niacin content in the algae samples tested by us ranged from 57.57 ± 1.70 μg/g (0.06 mg/g) to 514.82 ± 2.95 μg/g (0.52 mg/g). This is more comparable to the study of Edelmann et al. [69], where it was found to range from 0.14 mg/g to 0.28 mg/g in *Chlorella* and from 0.13 to 0.22 mg/g in *Spirulina*, with the lowest content recorded for *N. gaditana* (0.11 mg/g). In a study by Brown et al. [71], the vitamin composition of four Australian microalgae (*Nannochloropsis* sp., *Pavlova pinguis*, *Stichococcus* sp., and *Tetraselmis* sp.) was investigated. When expressed on a dry weight basis, the riboflavin content ranged from 25 to 50 μg/g. The vitamin content of *Nannochloropsis* sp. cultures showed marked differences, with a degree of variability similar to that observed among the four species grown in the 12:12 h light: dark regime and harvested during the late logarithmic phase. This study confirms that the content of B vitamins depends on the harvesting, extraction, and cultivation of microalgae.

### 3.6. Antioxidant Activity

#### 3.6.1. DPPH Assay

The antioxidant activity of ethanol extracts of microalgae was determined using the DPPH method (Table 5). The antioxidant activity ranged from IC_50_ 2.01 ± 0.04 to 0.44 ± 0.01 mg/mL, which is equivalent to 0.001475 0.006784 TEAC. Trolox was used as a standard, and the IC_50_ value for Trolox was 2.97 ± 0.79 μg/mL. The best antioxidant activity using the DPPH radical was obtained by the microalga *Nannochloropsis* sp., and in contrast, the weakest activity was found for the alga *Chaetoceros muelleri*.

#### 3.6.2. ABTS Assay

The antioxidant activity of the ethanolic extracts of algae was measured using ABTS assays (Table 6). The ability to scavenge ABTS radicals was determined from IC_50_ (mg/mL) of 0.39 ± 0.02 to 0.17 ± 0.01 corresponding to an equivalent from 0.006225 to 0.014891 TEAC, while the IC_50_ value for the Trolox standard was determined to be 2.48 ± 0.16 μg/mL. The best antioxidant activity was shown by *Nannochloropsis* sp. along with *Tisochrysis lutea*, and the weakest activity was found for the microalga *Tetraselmis chuii*. These results suggest that the overall antioxidant activity of the microalgae extracts is lower compared with the Trolox standard tested.

Studies have mainly focused on the ability of microalgae extracts to scavenge the radicals [2,2′-azino-bis(3-ethylbenzothiazoline-6-sulfonic acid), ABTS], 2,2-diphenyl-1-picrylhydrazyl (DPPH), peroxyl, and hydroxyl; however, other mechanisms of action have also been demonstrated, such as iron and copper chelation, ferric reducing antioxidant power (FRAP), and inhibition of lipid peroxidation [72]. In addition, the diversity of antioxidant activity of microalgae results from their diverse biochemical composition and ability to synthesize a broad spectrum of antioxidant chemicals [38]. Three strains of green microalgae, *ChlIorococcum* sp. C53, *Chlorella* sp. E53 and *Chlorella* sp. ED53, were investigated for their antioxidant properties [73]. For all strains, the ethanol extracts showed higher antioxidant activity compared with the hot aqueous extracts. In particular, the ethanolic extract of *ChlIorella* E53 had the highest DPPH radical scavenging activity of 68.18 ± 0.38% at 1.4 mg/mL (IC_50_ 0.81 mg/mL). Similarly, the study by Hajimahmoodi et al. [74] tested microalgae for antioxidant activity, and these microalgae also showed significant antioxidant power in the FRAP assay. The results were in agreement with those obtained from the DPPH assay.

### 3.7. Nutritional Use of Algae in the Food Industry

Microalgae have the capability to enrich the nutritional value of conventional food and feed, thereby benefiting both human and animal health [75]. In today’s market, consumers are increasingly looking for sophisticated, innovative, and nutritious food. Algae can be added to staple foods, such as bread, pasta, breakfast cereals, biscuits, crackers, dairy products, and desserts, and while enhancing their nutritional value, they can also alter the visual appeal of these foods by changing their color, microstructure, and rheological properties [76,77]. Bread is consumed commonly, which makes it ideal for delivering bioactive compounds from microalgae that have valuable health benefits. Various studies have investigated the use of microalgae and their extracts in the enrichment of bread, resulting in higher protein and mineral content. The effects on its color, crust, and crumb structure were analyzed. Microalgae have also found applications in gluten-free bread research, not only to improve its nutritional profile but also to mimic the gluten network, thereby improving the physico-chemical and rheological properties of the dough [77,78,79,80,81]. Lafarga et al. [82] produced crackers enriched in *Tetraselmis* sp. and *Nannochloropsis* sp., which were also studied in our work. Their findings indicated that the incorporation of microalgae led to increased polyphenol levels and the antioxidant capacity of the enzymatically digested extracts, suggesting a potential to produce healthier products. Fradique et al. [83] tested the potential to increase the nutritional value of pasta by incorporating microalgae into it. The microalgae-enriched pasta showed better quality parameters, such as color stability and firmness. It was also awarded higher acceptability scores than the control pasta due to its attractive color, texture, and nutritional value. Lafarga et al. [84] focused on the development of broccoli soups enriched with microalgae at concentrations ranging from 0.5% to 2.0% (*w*/*w*). The authors achieved an innovative microalgae-enriched soup characterized by increased antioxidant capacity and bioavailable polyphenol content. There are many possibilities to include small amounts of algae in products to improve their properties. Nonetheless, it is essential to assess the chemical properties of algae to confirm their suitability for use in the food industry, in addition to the commonly used spirulina and chlorella.

## 4. Conclusions

The growth of the global population and diminishing natural resources are significant drivers of development in the field of sustainable food production. The increasing demand for nutrients, particularly protein, coupled with the limitations of conventional agriculture, has led to the exploration of alternative raw materials. Microalgae emerge as a promising solution, exhibiting unique advantages, such as the ability to thrive in diverse environments without requiring arable land. This research focused on the characterization of commercially purchased microalgae grown under optimized conditions—*Nannochloropsis* sp., *Tetraselmis chuii*, *Chaetoceros muelleri*, *Thalassiosira weissflogii*, and *Tisochrysis lutea*. Through comprehensive analysis, we gained valuable insights into the diverse nutritional potential of these organisms. The findings revealed a substantial protein content ranging from 34.09% to 42.45%, with *T. weissflogii* exhibiting the highest percentage. Essential amino acids, crucial for human nutrition, were present in varying amounts across all the tested microalgae species. Glutamic acid emerged as the most abundant amino acid. Distinct mineral profiles were observed, with each microalgae species showcasing unique strengths. *T. chuii* showed an outstanding level of Ca and Mn, *C. muelleri* was the richest in Mg, Na, and Fe. *T. weissflogii* was found to contain the highest level of K, and in *T. lutea*, Cu, Zn, and Pb were the most abundant. The fatty acid analysis revealed differences in the levels of saturated fatty acids (SFAs), monounsaturated fatty acids (MUFAs), and polyunsaturated fatty acids (PUFAs) among the microalgae, with *T. lutea* standing out as a rich source of PUFAs. Furthermore, *T. lutea* was found to contain a diverse spectrum of polyphenolic compounds, including *p*-coumaric acid, catechin, chlorogenic acid, and *p*-hydroxybenzoic acid. The presence of these bioactive compounds suggests potential health benefits and justifies further research. The study also revealed significant amounts of B vitamins in the tested samples, with *T. lutea* particularly rich in niacin (B_3_) and riboflavin (B_2_). Antioxidant activity was confirmed for all tested microalgae by converting the IC_50_ values (mg/mL) from the DPPH and ABTS radical assays to Trolox equivalent antioxidant capacity (TEAC). These findings underscore the potential of microalgae as a biologically valuable resource. This potential is the result of their unique composition, inherent properties, simplicity in cultivation, and rapid biomass accumulation. The comprehensive nutritional analysis presented in this study contributes to our understanding of microalgae’s potential for applications in various fields, including human and animal nutrition, pharmaceuticals, and functional food. Further research and exploration of microalgae’s diverse attributes promise sustainable and innovative solutions to global challenges in nutrition and resource utilization.

## Figures and Tables

**Table 1:** Total protein content (%).

Microalgae	Content (%)
*Nannochloropsis* sp.	41.68 ± 0.58 ^ab^
*Tetraselmis chuii*	40.38 ± 0.27 ^b^
*Chaetoceros muelleri*	34.09 ± 0.39 ^c^
*Thalassiosira weissflogii*	42.45 ± 0.18 ^a^
*Tisochrysis lutea*	39.48 ± 1.80 ^b^

Values marked with the same superscript letter in columns do not differ significantly, *p* > 0.05.

**Table 2:** Minerals and trace elements contained in microalgae expressed in (µg/g).

Mineral	*Nannochloropsis* sp.	*Tetraselmis* *Chuii*	*Chaetoceros muelleri*	*Thalassiosira* *weissflogii*	*Tisochrysis* *lutea*
Ca	3350.00 ± 270.00 ^c^	54,900.00 ± 4390.00 ^a^	3340.00 ± 270.00 ^c^	2550.00 ± 200.00 ^d^	4840.00 ± 390.00 ^b^
Mg	3300.00 ± 260.00 ^c^	7350.00 ± 590.00 ^a^	7560.00 ± 600.00 ^a^	4180.00 ± 330.00 ^bc^	5210.00 ± 420.00 ^b^
K	10,100.00 ± 800.00 ^c^	12,600.00 ± 1000.00 ^c^	21,200.00 ± 1700.00 ^b^	34,700.00 ± 2800.00 ^a^	11,400.00 ± 900.00 ^c^
Na	13,900.00 ± 1100.00 ^c^	65,700.00 ± 5300.00 ^ab^	73,000.00 ± 5800.00 ^a^	49,900.00 ± 400.00 ^b^	54,800.00 ± 4400.00 ^b^
Cu	45.10 ± 3.60 ^c^	78.40 ± 6.20 ^a^	73.60 ± 5.90 ^ab^	68.80 ± 5.50 ^b^	78.50 ± 6.30 ^a^
Fe	605.00 ± 48.00 ^b^	898.00 ± 72.00 ^a^	969.00 ± 78.00 ^a^	580.00 ± 46.00 ^b^	307.00 ± 25.00 ^c^
Mn	94.50 ± 7.60 ^b^	139.00 ± 11.00 ^a^	93.80 ± 7.50 ^b^	106.00 ± 8.00 ^ab^	78.90 ± 6.30 ^c^
Zn	93.90 ± 7.50 ^bc^	106.00 ± 8.50 ^b^	103.00 ± 8.20 ^b^	83.5 ± 6.70 ^c^	181.0 ± 14.00 ^a^
Pb	56.90 ± 4.60 ^b^	69.50 ± 5.60 ^b^	94.80 ± 7.60 ^a^	94.8 ± 7.60 ^a^	108.0 ± 9.00 ^a^

Values marked with the same superscript letter in rows do not differ significantly, *p* > 0.05.

**Table 3:** Polyphenol profile composition (μg/g).

Sample	*Nannochloropsis* sp.	*Tetraselmis* *chuii*	*Chaetoceros muelleri*	*Thalassiosira* *weissflogii*	*Tisochrysis* *lutea*
Kaempferol	1.47 ± 0.02 ^b^	3.35 ± 0.05 ^a^	ND	0.99 ± 0.01 ^c^	ND
Vitexin	1.26 ± 0.03	ND	ND	ND	ND
Rutin	ND	1.03 ± 0.013 ^a^	ND	ND	1.07 ± 0.05 ^a^
*p*-Coumaric acid	ND	ND	ND	ND	0.36 ± 0.06
Catechin	ND	ND	ND	ND	0.79 ± 0.05
Chlorogenic acid	ND	ND	ND	ND	ND
Gallic acid	ND	ND	ND	ND	ND
*p*-Hydroxybenzoic acid	2.94 ± 0.07 ^c^	1.61 ± 0.10 ^d^	ND	10.96 ± 0.11 ^a^	7.28 ± 0.12 ^b^

Values marked with the same superscript letter in rows do not differ significantly, *p* > 0.05. ND—not detected.

**Table 4:** Content of B vitamins analyzed in microalgae expressed in (μg/g).

Sample	*Nannochloropsis* sp.	*Tetraselmis* *chuii*	*Chaetoceros muelleri*	*Thalassiosira* *weissflogii*	*Tisochrysis* *lutea*
Niacin (B_3_)	97.81 ± 1.21 ^d^	161.66 ± 2.04 ^c^	358.39 ± 2.42 ^b^	57.57 ± 1.70 ^e^	514.82 ± 2.95 ^a^
Riboflavin (B_2_)	2.44 ± 0.12 ^d^	8.27 ± 0.28 ^b^	6.94 ± 0.26 ^c^	ND	20.44 ± 1.96 ^a^
Folic acid (B_9_)	ND	ND	10.53 ± 0.92	ND	ND

Values marked with the same superscript letter in rows do not differ significantly, *p* > 0.05. ND—not detected.

**Table 5:** Antioxidant activity by DPPH assay.

Microalgae	IC_50_ (mg/mL)	TEAC Equivalent
*Nannochloropsis* sp.	2.01 ± 0.04 ^a^	0.001475 ^a^
*Tetraselmis chuii*	1.69 ± 0.02 ^a^	0.001748 ^a^
*Chaetoceros muelleri*	0.44 ± 0.01 ^a^	0.006784 ^a^
*Thalassiosira weissflogii*	0.92 ± 0.02 ^a^	0.003229 ^a^
*Tisochrysis lutea*	1.43 ± 0.02 ^a^	0.002076 ^a^

Values marked with the same superscript letter in rows do not differ significantly, *p* > 0.05.

**Table 6:** Antioxidant activity by ABTS assay.

Microalgae	IC_50_ (mg/mL)	TEAC Equivalent
*Nannochloropsis* sp.	0.39 ± 0.02 ^a^	0.006225 ^a^
*Tetraselmis chuii*	0.17 ± 0.01 ^b^	0.014891 ^b^
*Chaetoceros muelleri*	0.21 ± 0.01 ^b^	0.011897 ^c^
*Thalassiosira weissflogii*	0.34 ± 0.00 ^c^	0.007317 ^d^
*Tisochrysis lutea*	0.38 ± 0.03 ^a,c^	0.006588 ^e^

Values marked with the same superscript letter in rows do not differ significantly, *p* > 0.05.

## Data Availability

The original contributions presented in the study are included in the article, further inquiries can be directed to the corresponding authors.
